# Natural Infection of Pomegranate (*Punica Granatum*) by Apple Dimple Fruit Viroid

**DOI:** 10.3390/cells12010049

**Published:** 2022-12-22

**Authors:** Ana Belén Ruiz-García, Antonio Olmos, Armelle Marais, Chantal Faure, Thierry Candresse

**Affiliations:** 1 Insituto Valenciano de Investigaciones Agrarias (IVIA). Ctra, Moncada Náquera km 4.5, 46113 Moncada, Valencia, Spain; 2 UMR BFP, University of Bordeaux, INRAE, CEDEX 20032, 33882 Villenave d’Ornon, France

**Keywords:** pomegranate, fig, high-throughput sequencing, RT-PCR, natural host, apple dimple fruit viroid, plum viroid I

## Abstract

The analysis by high throughput sequencing (HTS) and RT-PCR of Spanish pomegranate fruits showing yellow rings revealed the presence of viroid isolates closely related to fig isolates of apple dimple fruit viroid (ADFVd). The analysis of pomegranate public RNASeq data (Sequence Reads Archives, SRAs) from Israel provided evidence for the presence of similar ADFVd isolates in pomegranate trees in this country. In addition, reads or contigs of plum viroid I (PVd-I) isolates were also identified in two of the analyzed SRA datasets from Israel, suggesting the presence of this second viroid in pomegranate. Full length ADFVd genomic sequences have been recovered, increasing knowledge on the diversity of this viroid and on the pomegranate virome in which only four viruses and one viroid had previously been reported.

## 1. Introduction

Pomegranate (*Punica granatum* L.) is an important crop in Spain covering 5619 ha with an estimated production of 84,477 metric tons in 2021 (MAPA, 2022) [https://www.mapa.gob.es/es/estadistica/temas/ (accessed on 1 August 2022)], with Spain being the main European producer. The south of the province of Alicante concentrates more than 75% of the Spanish production and hosts the Protected Designation of Origin (D.O.P.) Mollar de Elche. The knowledge of the virome of pomegranate is quite limited and, to date, only cucumber mosaic virus, tomato ringspot virus, grapevine leafroll associated virus 1, Passiflora edulis symptomless virus (PeSV) and hop stunt viroid have been reported as infecting this crop [1]. In Spanish pomegranates, only PeSV has been reported so far in plants that showed chlorotic spots along the leaf veins [2].

**Figure 1:**
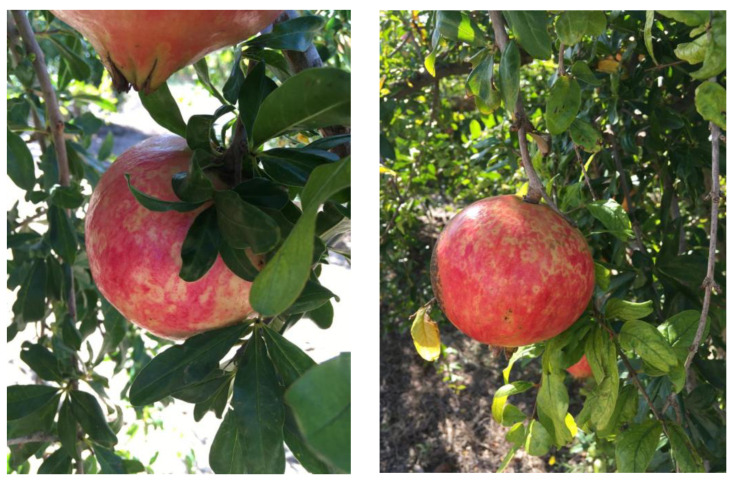
Yellow rings and arabesque discolorations observed on the fruits of the X1 (A) and X2 (B) pomegranate trees (cv. Mollar de Elche).

However, in summer 2019, during a routine inspection of the sanitary status of pomegranate trees cv. Mollar de Elche in one of the main production areas of Spain (Elche), fruits showing bright yellow rings were observed (Figure 1). There were no internal symptoms in those fruits or leaf symptoms on the plants bearing them. A fruit with similar symptoms and coming from the same production area had previously been identified in a French supermarket. To investigate the etiology of these symptoms and the possible presence of viruses or viroids, nucleic acids extracted from fruits and leaves from two trees with symptomatic fruits and from the symptomatic fruit obtained from the French supermarket were analyzed by high-throughput sequencing (HTS). The results revealed, in all of them, the presence of variants of apple dimple fruit viroid (ADFVd), a member of the genus *Apscaviroid* in the *Pospiviroidae* family. ADFVd was first identified as a viroid causing a disease characterized by fruit deformations of variable severity in several apple cultivars [3]. Natural infection of fig (*Ficus carica* L.) by variants of ADFVd forming a distinct phylogenetic cluster has also been reported [4]. To date, the genus *Apscaviroid* contains 18 members and is the largest viroid genus [5]. Following a recent re-evaluation of species demarcation criteria in viroid taxonomy [6], the current molecular criterion in the *Apscaviroid* genus is less than 78% nucleotide identity between isolates belonging to different species [5].

Here, we show the potential of HTS for the analysis of samples without previous knowledge of any viral genomic sequence, and in addition to the identification of ADFVd variants in the Spanish samples, identify variants of plum viroid I from Sequence Reads Archives (SRAs) of pomegranate trees cultivated in Israel. This data improves knowledge of the pomegranate virome and, consequently, has the potential to improve sanitary selection and certification programs in pomegranate.

## 2. Materials and Methods

### 2.1. Plant Material

Leaves and fruit pedicels from two pomegranate trees (X1, X2) cv. Mollar de Elche showing yellow ring discolorations on their fruits (Figure 1) were collected during the late 2019 summer in Elche, Spain. In addition, a fruit (X3) showing similar discolored rings was obtained in a French supermarket. This fruit, of an unknown variety, could be traced back to the same Spanish production area.

### 2.2. Sample Preparation and HTS Analysis

In the case of the X1 and X2 samples, total RNA was purified from 200 mg of leaves and fruit pedicels from each sample using the Plant/Fungi total RNA purification kit (Norgen Biotek Corporation, Thorold, ON, Canada) according to the manufacturer’s protocol. The library was prepared using the TruSeq Stranded Total RNA LT Sample Prep Kit. RNA quality control, library construction and sequencing using the NextSeq 500 platform (paired 2 × 75 nt) were performed at Biopolis (Valencia, Spain).

In the case of sample X3, small RNAs (sRNAs) were purified using the mirPremier microRNA isolation kit (Sigma-Aldrich, St. Louis, MO, USA) from 200 mg of fruit pedicel according to the manufacturer’s protocol. The library was prepared using TruSeq Small RNA Library Kit. RNA quality control, library construction and sequencing using a HiSeq 2500 platform were similarly performed at Biopolis (Valencia, Spain).

### 2.3. Bioinformatic Analysis of HTS Data

For RNASeq analyses, quality control and trimming were performed using CLC Genomics Workbench v.10.1.1 (Qiagen Bioinformatics, Hilden, Germany). Host genome subtraction was performed using the pomegranate reference genome (GCF_007655135.1_ASM765513v2) and chloroplast genome (NC_035240.1). De novo assembly was carried out with the same software and recovered contigs were annotated by Blast analysis (BlastN/X) against local and Genbank virus, viroids and nt/nr databases. Contigs were extended by rounds of mapping of residual unassembled reads using Geneious Prime 2020 (Biomatters, Inc., Auckland, New Zealand).

For sRNA analysis, quality control and trimming were performed using FastQC 0.11.5 (https://www.bioinformatics.babraham.ac.uk/projects/fastqc/ (accessed on 1 August 2022)) and prinseq-lite-0.20.4. (https://github.com/uwb-linux/prinseq (accessed on 1 August 2022)). De novo assembly was performed using Vague 1.0.5 [7], a Velvet assembler graphical front end, using 11, 13, 15, 17 and 21 k-mers. Contigs were annotated as indicated above for RNASeq analysis and extended with Bowtie 2 implemented in Geneious Prime 2020.

The screening of 31 representative pomegranate public RNASeq datasets (GenBank Sequence Reads Archives, SRA) for the presence of ADFVd was performed by BlastN analysis using the complete genome of a pomegranate ADFVd isolate as the query. SRAs for which ADFVd reads were identified were downloaded and analyzed using CLC Genomics Workbench as described above.

### 2.4. RT-PCR Analysis

ADFVd presence was confirmed by RT-PCR amplification and Sanger sequencing of the obtained amplicons. The specific primer pairs used were designed based on the sequence of pomegranate isolates: ADFVd F3: 5′-TTGACTAGATGCCCGCCTGAC-3′ and ADFVd R3 5′-CACGACCTAGAGCCGCCTCCA-3′ or ADFVd F4: 5′-AAGGACTTGACTAGATGCCCGCCTG-3 and ADFVd R4: 5′-AGTCATTAGCGCAGGGGGGTGC-3′. Amplifications were either performed on purified double-stranded RNA extracts or on total nucleic acids purified using a guanidine thiocyanate—silica capture method described as protocol 2 in [8]. Complementary DNA was synthetized using the RevertAid First Strand cDNA Synthesis Kit (ThermoFisher Scientific, Illkirsch, France) and the PCR reaction performed using 0.4 μM of each primer, the Advantage GC Genomic LA Polymerase Mix (Takara, St Germain en Laye, France) and 2.5 μL of cDNA. The cycling scheme for the ADFVd F3-R3 primer pair consisted of a denaturation step at 94 °C for 1 min, 40  cycles of 94 °C for 30 s, 55 °C for 30 s and 72 °C for 30 s with a final elongation step at 72 °C for 3 min. For the ADFVd F4-R4 primer pair amplification conditions included a denaturation step at 94 °C for 1 min, 35 cycles of 94 °C for 30 s, 65 °C for 30 s and 72 °C for 30 s with a final elongation step at 72 °C for 3 min. Amplicons were Sanger-sequenced in both orientations after purification using the mi-PCR Purification Kit (Metabion International AG, Martinsried, Germany) following the manufacturer’s instructions.

### 2.5. Sequence Comparisons and Phylogenetic Analyses

Comparison of pomegranate ADFVd sequences obtained in this study with representative isolates from apple and fig retrieved from GenBank was performed using a multiple alignment of the complete genomes obtained using ClustalW implemented in MEGA X [9]. Phylogenetic trees were reconstructed using the Maximum Likelihood algorithm implemented in MEGA X applying the Kimura 2-parameters substitution model and 500 bootstrap replicates.

## 3. Results

### 3.1. Symptoms on Fruits

Following an initial observation of a pomegranate fruit (X3) with virus-like symptoms in the form of bright yellow rings and arabesque discolorations, a survey of plants showing similar symptoms in the production area to which this fruit had been traced back was performed in 2019. Of 74 pomegranate trees surveyed, 7 trees bearing fruits with similar discolored rings (Figure 1) were identified. Of these, 2 trees, X1 and X2, together with the initial X3 fruit, were used for HTS-based virome analysis.

### 3.2. HTS Analysis of Pomegranate Samples and Identification of ADFVd Variants

The analysis of the RNASeq dataset obtained from sample X1 yielded 5,401,062 paired-end reads (average size 60.4 nt) after quality control, adapters trimming and pomegranate genome subtraction. De novo assembly generated 1775 contigs and revealed the presence of PeSV (17 contigs of between 223 nt and 450 nt) and the complete genome of a variant of ADFVd (contig 24.1, 311 nt long, recovered by assembling a total of 796 reads for a mean coverage of 127.7×).

A similar analysis performed on the dataset from sample X2 (24.2) yielded 6,385,092 paired-end reads (average size 59.5 nt) after quality control, adapters trimming and pomegranate genome subtraction. A total of 4396 contigs were de novo assembled and their annotation allowed to identify a single contig representing the complete genome of another variant of ADFVd (contig 24.2, length 309 nt, integrating 516 reads for a mean coverage of 82.9×). No contigs related to PeSV or to any other plant virus or viroid were additionally identified by BlastN or tBlastX.

A total of 573,352 reads ranging from 15 nt to 75 nt was obtained from purified small RNAs from sample X3. De novo assembly using Velvet with a k-mer of 13 yielded 481 contigs (length between 50 and 156 nt) and with a k-mer of 15 yielded 581 contigs (length between 50 and 210 nt). In the first case, two contigs (101 nt and 55 nt) related to ADFVd were identified, while three ADFVd contigs (77 nt, 69 nt and 41 nt) were obtained in the second case. Scaffolding and extension of contigs allowed the recovery of a partial ADFVd genome sequence of 257 nt (integrating 165 reads for a mean coverage of 14.9×). The gap was closed by Sanger sequencing of a RT-PCR amplicon, allowing to confirm ADFVd presence in the X3 sample and to determine the complete genome sequence of the ADFVd variant present in the X3 sample. The presence of ADFVd in the X3 fruit was validated by RT-PCR amplification using the ADFVd F3-R3 and the ADFVd F4-R4 primer pairs on purified double-stranded RNAs and total RNAs extracted from the fruit rind. Amplicons of the expected size were obtained (with better performance for the F4-R4 primer pair, which yielded more intense amplicon bands) and the sequence assembled from the amplicons found to be identical to the HTS-derived X3 sequence.

The sequences of the various ADFVd isolates determined from the X1, X2 and X3 samples have been deposited in GenBank under the OP807947–49 accession numbers.

### 3.3. Datamining of Public Pomegranate RNAseq Data

In an effort to see whether other cases of ADFVd infection in pomegranate could be identified, a datamining effort in public pomegranate transcriptome RNASeq data was undertaken. A selection of 31 representative pomegranate SRAs was identified by selecting non-redundant datasets, i.e., datasets generated by different laboratories or countries or involving different pomegranate varieties (Supplementary Table S1). BlastN analysis of these 31 pomegranate SRAs using the full genomic sequence of a pomegranate ADFVd variant as the query revealed the presence of ADFVd in two pomegranate SRAs: SRX5386154, an Illumina RNASeq dataset from the P.G.116-17 variety obtained in Israel, and SRX5386156, an Illumina RNASeq dataset also generated in Israel but from the P.G.200-211 variety. In a third dataset, SRX395468, corresponding to a 454 pyrosequencing EST sequencing project for the P.G.127-28 variety in Israel, a single divergent EST was identified.

The three datasets were downloaded, imported in CLC Genomics Workbench and de novo assembled before contigs annotation by BlastN to identify those with ADFVd affinities. The results obtained are summarized in Table 1.

As can be seen in Table 1, besides the 454 single EST identified in SRX395468, one viroid contig representing a full-length genome with a low 4.2x average coverage, could be assembled from SRX5386154. From the third SRA, SRX5386156, two viroid contigs could be assembled, representing full-length viroid genomes, both of them with deeper 23x-37x coverage. More detailed comparisons indicated that the viroid contig from SRX5386154 and Contig2 from SRX5386156 are related to the ADFVd pomegranate variants from samples X1, X2 and X3 with nucleotide divergence levels with them of 2.6 and 10.4%. Surprisingly, the SRX395468 EST and Contig1 from SRX5386156 had much closer relationships with another viroid, plum viroid I (PVd-I) [10], with between 14.6 and 15.8% divergence with the reference sequence for that viroid (MN734737). The sequences of the various ADFVd and PVd-I isolates identified by datamining have been deposited in GenBank under the BK062881–84 accession numbers. Despite the fact that HSVd infection has been reported in pomegranate, we did not find evidence of HSVd presence in the screened SRAs, but given that their number is limited, it cannot be assumed that analyzing further pomegranate datasets will not reveal HSVd presence.

### 3.4. Phylogenetic Analysis

**Figure 2:**
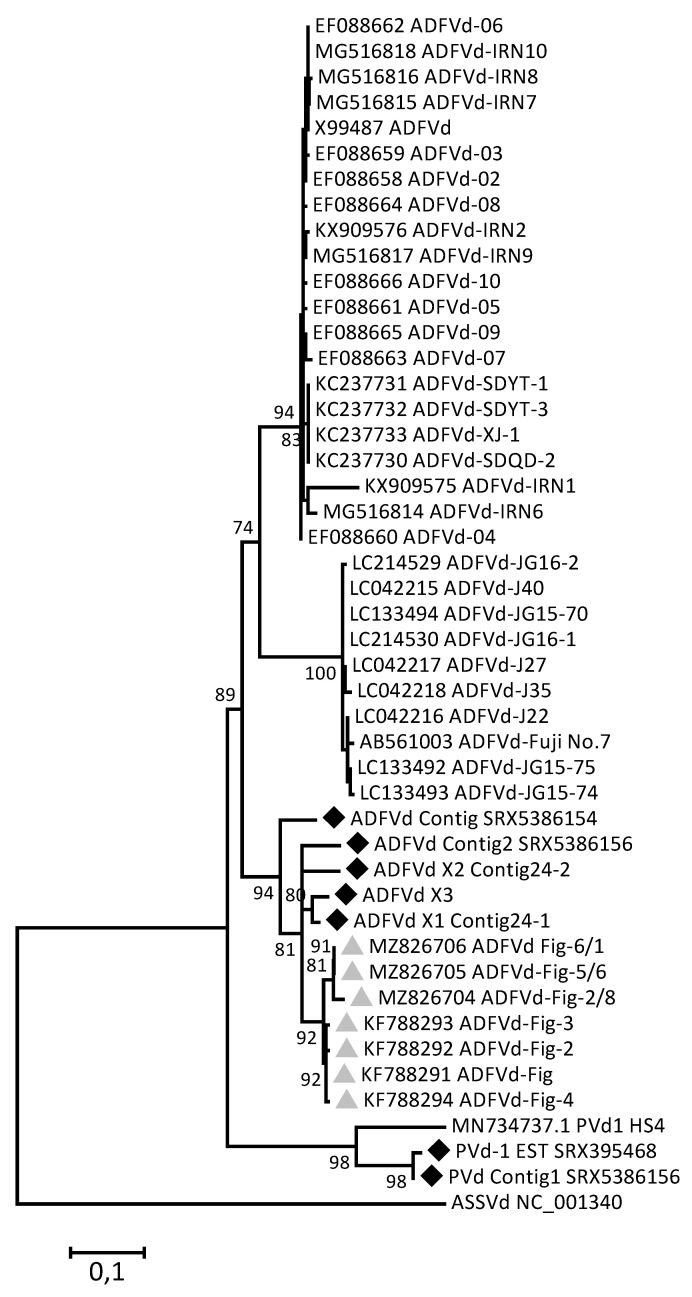
Maximum likelihood phylogenetic tree reconstructed from a multiple alignment of complete genome sequence of ADFVd and PVd-I isolates sequences. The sequences determined in the present work are marked by a black diamond while the ADFVd sequences from fig are marked by a grey triangle. All other isolates are from apple, the only other known host of ADFVd. Accession numbers are provided for sequences retrieved from GenBank. The scale bar indicates 10% nucleotide sequence divergence. Only bootstrap values higher than 70% are shown.

A phylogenetic analysis of the ADFVd and PVd-I variants identified either through HTS or datamining efforts was performed, including all ADFVd isolates available in GenBank irrespective of their host of origin. The maximum likelihood tree presented in Figure 2 unambiguously demonstrates that the pomegranate and fig variants of ADFVd cluster together with high bootstrap support, in a separate group from all ADFVd apple isolates. In parallel, the SRX395468 EST and Contig1 from SRX5386156 cluster with strong bootstrap support with the reference PVd-1 sequence, confirming their identification as PVd-I isolates.

## 4. Discussion

This study improves our knowledge of the pomegranate virome and represents the first identification of a natural ADFVd infection in this crop. Multiple ADFVd detections from HTS data of Spanish samples and from the RNASeq SRAs from Israel confirm ADFVd infection in a range of pomegranate samples. This is further confirmed by RT-PCR detection followed by Sanger sequencing of the amplicons obtained from the X3 fruit obtained from a French supermarket.

The phylogenetic analysis of a multiple alignment of ADFVd complete genome sequences reveals that pomegranate and fig isolates are closely related, which suggests that isolates may possibly be exchanged between these two crops that can frequently be found growing close to each other in Mediterranean countries. This could, for example, occur during pruning of the trees using the same tools. The close relationships between fig and pomegranate isolates separate them from the more divergent isolates found in apple, a crop that is less expected to be found growing side-by-side with either fig or pomegranate. In addition, the presence of PVd-I isolates in SRAs from Israel suggests that this viroid could also be part of the pomegranate virome.

A causal relationship between ADFVd infection and the observed fruit symptoms is a distinct possibility given that no other viral pathogen was identified from the HTS sequencing reads obtained from the X2 plant or from the X3 fruit. However, a causal role of ADFVd in the observed yellow ringspot symptoms will clearly need to be further supported by experimental transmission work, the inoculation of ADFVd infectious clones or further surveys allowing to strengthen the limited correlation established here between symptoms and ADFVd presence. The RT-PCR assay developed here could prove useful to clarify this issue.

The datamining efforts in pomegranate SRAs unexpectedly provided evidence suggesting that, in addition, to HSVd and ADFVd, PVd-I might naturally infect pomegranate. However, given that the original pomegranate materials involved could not be accessed, it was not possible to experimentally confirm this finding. It should, therefore, be considered with caution and not be considered as established evidence. Further efforts are clearly needed to validate or dispel a PVd-I host status for pomegranate and evaluate the potential impact of PVd-I on this valuable crop.

## Figures and Tables

**Table 1:** Properties of the analyzed pomegranate SRAs and of the viroid EST/contigs identified.

SRA	Variety/Country	Total Reads	Contig/EST	Reads/Coverage	Identity
SRX395468	P.G.127-28/Israel	728,664	EST	1/1×	PVd-I
SRX5386154	P.G.116-17/Israel	121,705,906	Contig	13/4.2×	ADFVd
SRX5386156	P.G.200-211/Israel	121,357,374	Contig1	194/37×	PVd-1
			Contig2	118/23×	ADFVd

## Data Availability

The genome sequences of ADFVd and PVd-1 isolates determined in the present study have been deposited in GenBank under the OP807947-49 and BK062881-84 accession numbers.

## Supplementary Materials

The following supporting information can be downloaded at: https://www.mdpi.com/article/10.3390/cells12010049/s1, Table S1: Information on the various pomegranate SRAs used for datamining.
